# Exploring preferences for domain-specific goal management in patients with polyarthritis: what to do when an important goal becomes threatened?

**DOI:** 10.1007/s00296-015-3336-8

**Published:** 2015-08-12

**Authors:** Roos Y. Arends, Christina Bode, Erik Taal, Mart A. F. J. Van de Laar

**Affiliations:** Department of Psychology, Health and Technology, Arthritis Centre Twente, University of Twente, P.O. Box 217, 7500 AE Enschede, The Netherlands; Department for Rheumatology, Arthritis Centre Twente, Medisch Spectrum Twente, Enschede, The Netherlands

**Keywords:** Adaptation, Threatened personal goals, Patient-perspective, Coping, Vignette, Rheumatoid arthritis

## Abstract

Usually priorities in goal management—intended to minimize discrepancies between a given and desired situation—are studied as person characteristics, neglecting possible domain-specific aspects. However, people may make different decisions in different situations depending on the importance of the personal issues at stake. Aim of the present study therefore was to develop arthritis-related vignettes to examine domain-specific goal management and to explore patients’ preferences. Based on interviews and literature, situation-specific hypothetical stories were developed in which the main character encounters a problem with a valued goal due to arthritis. Thirty-one patients (61 % female, mean age 60 years) evaluated the face validity of the newly developed vignettes. Secondly, 262 patients (60 % female, mean age 63 years) were asked to come up with possible solutions for the problems with attaining a goal described in a subset of the vignettes. Goal management strategies within the responses and the preference for the various strategies were identified. The 11 developed vignettes in three domains were found to be face-valid. In 90 % of the responses, goal management strategies were identified (31 % goal maintenance, 29 % goal adjustment, 21 % goal disengagement, and 10 % goal re-engagement). Strategy preference was related to domains. Solutions containing goal disengagement were the least preferred. Using vignettes for measuring domain-specific goal management appears as valuable addition to the existing questionnaires. The vignettes can be used to study how patients with arthritis cope with threatened goals in specific domains from a patient’s perspective. Domain-specific strategy preference emphasizes the importance of a situation-specific instrument.

## Introduction

Chronic conditions present a set of challenges to patients and their families who must endure behavioral and psychological changes. Patients have to deal with disease symptoms, increasing disability, emotional impact, complex medical regimens, lifestyle adjustments, and securing helpful medical care [1]. As a result of the changes that the disease entails, important personal goals may be threatened or even unachievable [2–4]. In addition to the emotional impact of the disease and associated challenges, unreachable or threatened goals may have a negative influence on well-being. Although lower levels of well-being are found in patients, not all patients experience lower well-being, and, in fact, a substantial number of patients evaluate their life as meaningful [5–7]. As coping can improve adaptation to the above-mentioned challenges and, consequently, increase well-being, knowledge of appropriate coping strategies facilitates well-being for those who struggle with finding a (new) balance in living with a chronic condition.

A way to cope with threatened personal goals is by using goal management which attempts to minimize discrepancies between the goals of a person and the actual situation [8, 9]. However, the distinction between coping from a dispositional perspective as opposed to a contextual perspective is a dichotomy among coping theorists [10, 11]. These perspectives contain contrasting underlying determinants of the coping process. Applying the dispositional and contextual perspectives to goal management, the difference is whether the applied mode of goal management is determined by stable trait characteristics of a person or by situation-specific factors. A useful integration of both perspectives can be found in the model of Moos and Holahan [10], which emphasizes that individuals are active agents who can shape the outcomes of stressful life circumstances and, in turn, be shaped by them.

Existing questionnaires about goal management are designed to measure general tendencies. A series of statements is presented to participants, who are asked to indicate to what degree a statement describes their typical reaction pattern. As the questionnaires measure dispositional goal management, they gather information on how a person judges his or her own behavior in general. However, reflecting the contextual perspective on coping, people may make different decisions in different situations depending on the importance of the personal issues at stake. Little is known about the choices that people make when confronted with limitations and declining ability to perform valued activities in specific domains. A domain-specific measurement method can be applied for this purpose. Additionally, the use of questionnaires can raise ambiguity as respondents are asked to make decisions and judgments from abstract and limited information [12]. It remains, for example, unclear whether a respondent was thinking of a particular goal, occurrence, or time period when responding to the statements.

Hypothetical scenarios or vignettes that describe arthritis-specific situations might be a promising method to collect information on goal management in polyarthritis patients. Vignettes are valued as a method to measure attitudes, beliefs, and values, especially about abstract concepts related to health and illness [13, 14]. The use of vignettes helps to standardize stimuli across respondents [12], making it a convenient and expedient method for collecting extensive amounts of data from large samples [13]. Vignettes should contain valid and typical situations that are recognizable by the majority of respondents. In that way, the reaction to the vignette is more comparable with natural daily situations.

Almost two million adults in the Netherlands are diagnosed with a rheumatic disease. In this group, 420,000 people have a form of inflammatory arthritis [15]. Medical management may alleviate inflammation and part of the pain, but for many patients fluctuating pain, fatigue, disability, deformity, and reduced quality of life persists [16, 17]. Disease symptoms like pain, fatigue, and functional limitations can make it difficult and even impossible to attain goals in important life domains [18].

Studies from two different but complementary approaches offer insights into the life domains that are influenced by arthritis. One approach includes studies that researched domains from a professional/caregiver, decision-maker, and/or epidemiological perspective, e.g., [19–24]. Limitations in physical and mental functioning, activities, and participation were reported [23], and domains influenced by arthritis were specified as: work and remunerative employment; recreation and leisure; family and social or intimate relationships [19, 21, 23, 25–27]. Limitations in one domain can have significant impact in other domains of life. For example, polyarthritis has been demonstrated to negatively influence participation and work ability [21, 25, 28], possibly resulting in loss of family income, status, and social support [28].

The second approach is reflected in studies that researched the patient perspective of the impact of the disease on daily life. Research methodologies are diverse, ranging from: clinical case reports [29], interview studies using (life) stories of patients [30–32], the use of focus groups [33], cohort studies using structured interviews [18, 34, 35], and literature reviews [36]. Some of these patient-perspective studies revealed problems with attaining or maintaining goals in both private and public domains of life, including work, social relationships, leisure activities, and domestic tasks [2, 37]. Most of the previous mentioned studies, however, focused on what patients reported as important concepts, general outcomes of treatment, or adjustments made to life. Examples of such reports are: “feeling well in myself,” “being normal again,” “fatigue,” and “emotional consequences” [33, 36]. From studies based on both the approaches of professional perspective and the patient-perspective studies, one can conclude that arthritis has an influence on a wide variety of life domains of patients which, therefore, might be useful to distinguish.

Changes in life domains caused by a chronic disease can have psychological and social consequences for patients and can affect their identity [38]. To have and strive for personal goals are important for well-being [39, 40], while the inability to achieve goals can cause frustration and depression. The loss of activities in some domains appears to be more closely linked to an increase in depressive symptoms than the loss of activities in other domains [41]. For example, declines in the ability to perform recreational activities and engage in social interactions were found in the longitudinal study of Katz et al. [41] to be linked to the onset of depressive symptoms. In particular, when the goals are closely linked to the identity of a person, unattainable goals can have a negative influence on well-being. Several studies showed that among rheumatoid arthritis (RA) patients, there is a higher prevalence of anxiety and depressive symptoms and lower levels of purpose in life than in healthy controls [42–44]. Psychosocial problems, in turn, can have an adverse influence on disease burden. Patients experiencing psychosocial problems report higher disease scores and more pain, even though they do not have higher disease activity or lower functional ability than other patients [45].

To find an equilibrium between which goals to maintain and which to disengage from may be a beneficial process to sustain well-being. This implies being flexible and able to react to obstacles to personal goals in various ways [4, 46, 47]. People can use several strategies when they encounter an obstacle on their path to a goal. These goal management strategies are intended to minimize discrepancies between the given situation and the desired situation. Ideally, patients would weigh possible strategies against their own potential and constraints from the environment. Individuals require a repertoire of strategies and skills to successfully choose and apply the strategies in every particular case of a threat to a goal.

Several goal management strategies are described in the literature. The integrated model of goal management [4] combines four strategies from the dual process model of assimilative and accommodative coping [8, 9, 48] and the goal adjustment model [49]. The strategies in this model are as follows: (1) Goal maintenance, implying active attempts to alter unsatisfactory life circumstances and situational constraints in a way that fits personal preferences. (2) Goal adjustment, the revision of self-evaluative standards and personal goals in accordance with perceived deficits and losses to make the situation appear less negative or more acceptable. (3) Goal disengagement, the withdrawing of effort and commitment from a goal that is perceived as unattainable. (4) Goal re-engagement, the identification, commitment to and pursuing of new goals, in addition to or instead of other goals.

The overall objective of our study was to examine domain-specific goal management in arthritis patients. To reach this objective, we conducted two studies. The first was to develop vignettes that reflect a realistic situation in which a valued goal of an arthritis patient is threatened. The vignette instrument—consisting of situation-specific hypothetical stories—examines contextual or domain-specific goal management in polyarthritis patients and expands existing questionnaires. Use of both measures in future research may facilitate the understanding of how adaptive coping moderates the influence of stressors on well-being. Our second objective was to use the vignettes to study the goal management strategies that patients create and prefer when presented the arthritis-specific situations in the vignettes. To study the applicability of the integrated model of goal management in practice, the strategies from this model were used to categorize the answers provided by respondents and to investigate whether these strategies capture the provided reactions.

## Methods

Our objective was to develop a pool of vignettes that could be applied to several situations and populations (Part 1). The vignettes should contain threatened goals of arthritis patients specific to domains that may be affected by arthritis. Arthritis patients should assess the vignettes as recognizable and realistic. After the vignettes were composed and evaluated, a subset of vignettes was chosen to study patients’ reactions to the vignettes (Part 2). Our interest in this second part was mainly the applicability of the vignettes to study goal management strategies of arthritis patients. For this purpose, we chose the most generic vignettes for our subset, as not all vignettes were relevant and applicable for this sample of arthritis patients. In Part 2, we had the following questions: (1) Are the four goal management strategies; goal maintenance, goal adjustment, goal disengagement, and goal re-engagement, recognizable in the answers? (2) Are the four strategies exhaustive? (3) Do the strategies that the respondents mention and prefer differ between the domains? In addition, we added an “open vignette”, in which respondents were asked to describe one of their own situations in which a goal was threatened due to arthritis. This additional vignette was used to study, in an explorative way, the themes and domains people mentioned. The study was approved by the internal review board of the Faculty of Behavioural Sciences at the University of Twente.

### Part 1: development of vignettes

#### Development

To identify the vignette topics, interviews with patients with RA about coping with arthritis and with threatened personal goals [50] and literature on limitations and threatened domains experienced by arthritis patients were used. Eleven hypothetical stories in which the main character encounters a problem with a valued goal due to arthritis were formulated. The wording and use of language of the vignettes was initially tested in a small pilot study. There were no difficulties regarding the wording, language and understanding of the vignettes. Only small adjustments were made in sentence structure.

#### Sample

Participants of the “Arthritis Research Partners” forum of the Arthritis Centre Twente were invited to participate in testing the feasibility of the vignettes. This forum consists of voluntary participants who have a rheumatic condition for at least 2 years and are willing to cooperate in research. Invitation letters were sent to 40 forum participants, and after a week, people were contacted by telephone. Thirty-two persons were willing to participate (response rate 80 %).

#### Participants

Thirty-one persons with RA participated in a questionnaire study (61 % female, mean age 59.5 years). Demographics of the participants are shown in Table 1 (Part 1). One person was excluded due to too much missing data.

**Table 1:** Demographic characteristics of the participants

Demographic characteristics	Part 1: development	Part 2: goal management strategies
Sex, *n* (%)	31	262
Male	12 (38.7)	105 (40.1)
Female	19 (61.3)	157 (59.9)
Age (years), mean (SD), range	59.5 (13.2), 33–83	62.8 (11.7), 33–90
Marital status, *n* (%)		
Not living with partner/no partner	7 (22.6)	61 (23.3)
Living with partner	24 (77.4)	196 (74.8)
Missing data	0	5 (1.9)
Educational level, *n* (%)^a^		
No/lower	4 (12.9)	96 (36.7)
Secondary	19 (61.3)	109 (41.6)
Higher	8 (25.8)	51 (19.4)
Missing data	0	6 (2.3)
Work status, *n* (%)		
No paid job	18 (58.1)	179 (68.3)
Full-time and part-time employment	13 (41.9)	79 (30.1)
Missing data		4 (1.5)
Disease duration (years), mean (SD)	13.3 (11.1)	15.9 (12.2)
Comorbidities, *n* (%)/mean (SD)^b^	17 (54.8)	1.6 (1.5)
Pain, mean (SD)^c^	N/A	4.11 (2.4)
HAQ-DI^d^, mean (SD)	N/A	.97 (.7)

^a^Low: no education, primary school, or lower vocational education; middle: high school and middle vocational education; high: high vocational education and university

^b^Comorbidities were measured in different ways in the two studies

^c^Amount of pain in the past week: 1 = not at all—10 = unbearable

^d^HAQ-DI: measures functional limitations in arthritis patients [55]

#### Procedure and questionnaire

Participants could participate in the study either at home or at the university, in the presence of a student–assistant. Participants were asked to read and answer the vignettes and subsequently answer seven written questions regarding the vignettes regarding the face validity and understandability of the vignettes. Examples include whether participants had understood the vignettes, whether they found the vignettes realistic, and whether the impact of RA on their life as portrayed in the vignettes was personally recognizable (questions appear in Table 4). A five-point Likert scale was used, with 1 = totally disagree and 5 = totally agree. Also the spontaneous reactions of participants after reading the vignettes were collected and content-analyzed.

### Part 2: goal management strategies in response to a subset of vignettes

#### Sample and recruitment

For the second study, the vignettes were included in a larger questionnaire study. For more details on design and methods, see Arends et al. [4]. The study consisted of three measurement waves. Participants were randomly selected from the electronic diagnosis registration system of an outpatient clinic for rheumatology. The following inclusion criteria were applied to select participants: (1) patient is diagnosed with polyarthritis and (2) patient is receiving treatment for polyarthritis. After initial selection, the rheumatologists checked the charts for the additional inclusion criteria: (3) patient is 18 years or older and (4) patient is able to complete the questionnaire in Dutch, either autonomously or with help from a relative. Out of 803 patients, 636 patients met the inclusion criteria and received an invitation letter, questionnaire, and informed consent form. Information on demographics, goal management, indicators of adaptation to a chronic disease, and disease characteristics was collected. In the third measurement wave that contained the vignettes, 262 patients participated (59.9 % female, mean age 62.8 years). Demographic and clinical characteristics of respondents are shown in Table 1 (Part 2).

#### Vignettes

**Fig. 1:**
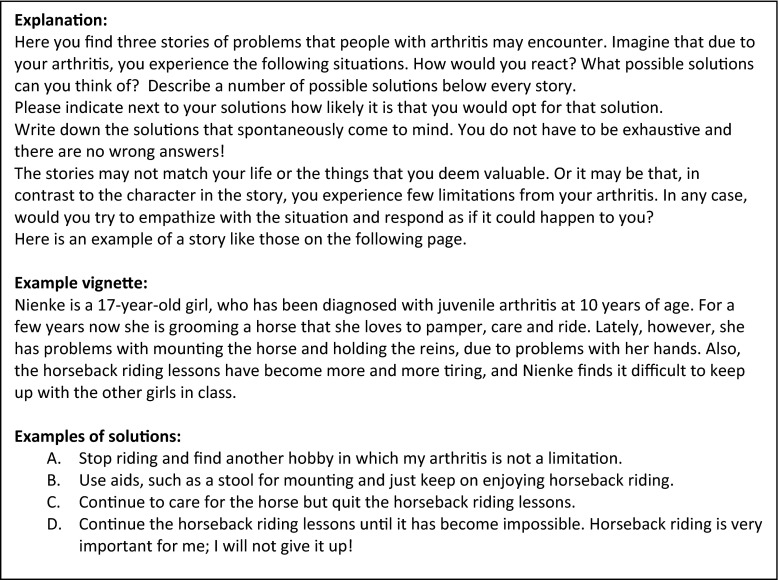
Introduction and example vignette

**Fig. 2:**
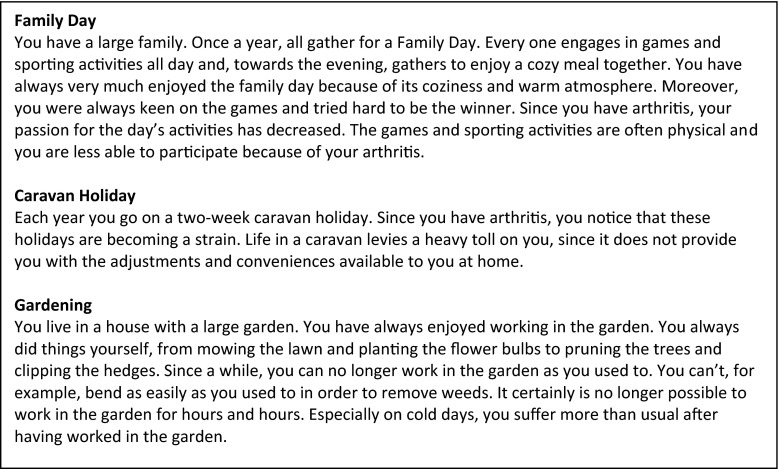
Subset of vignettes about Family Day, caravan holiday, and gardening

The vignettes were included at the end of the questionnaire. The exact (translated) wording of the introduction for the vignettes appears in Fig. 1. First an example vignette was given along with possible answer options for that particular vignette. The example vignette was specifically written for this purpose and does not stem from the earlier described study on the development of the vignettes. Subsequently, three vignettes from different life domains are presented (Fig. 2). The first vignette is from the social domain—the main character experiences problems with participating in the annual Family Day games and sports due to physical pain. (In the Netherlands, a Family Day is usually a day where activities are organized for the extended family to strengthen their relationships). The second vignette deals with problems in the leisure activities domain. Due to the unavailability of adjustments and facilities, the main character experiences problems during vacation with the caravan. The third vignette deals with the domain of independent functioning. Due to physical pain, the main character has difficulties working in the garden. In addition, we asked people to describe one of their own (current or past) situations in which they experienced problems in attaining a personal goal. For every vignette, participants were asked to answer the following two questions: (1) *What possible solutions can you come up with for the problem described above*? (to a maximum of six solutions) and (2) *How likely is it that you would try this solution?* Participants were then asked to rate their own described solutions on a scale from 1 (I would *absolutely* try this) to 5 (I would *never* try this).

#### Analysis of responses

A detailed codebook was developed in discussion rounds between two authors (ET and RYA). The codebook contained a description of the strategies and examples of answers per vignette (see Table 2 for examples). The same two authors separately coded 10 % of the answers for every vignette. For two vignettes, a sufficient degree of agreement was reached after the first coding round. Based on the consensus of the authors, the encodings of the remaining vignette (Family Day) were discussed again and the codebook was clarified. Another 10 % of the responses on this vignette were coded by the same two authors and then a sufficient degree of agreement was reached for this vignette as well (see Table 2). The open vignette was content-analyzed to study, in an explorative way, the themes and domains people mentioned.

**Table 2:** Definitions of strategies, examples of answers, and degree of similarity per vignette

	Description	Vignette 1: Family Day		Vignette 2: caravan holiday		Vignette 3: gardening	
		Example of solution	Kappa^a^	Example of solution	Kappa	Example of solution	Kappa
Maintenance of goals	Active attempts to adjust the environment so that your goal is attainable	Try to join all day and accept the setback I get later	.59	Raise the bed by using a higher mattress	.72	Spread the work over several days	.91
Adjustment of goals	Adjust the goal to what is feasible, given the situation	Participate in everything but less fanatically	.79	Sell the caravan and spend the holidays in a hotel	.79	Ask someone else to do the heavy work	.71
Disengagement of goals	Withdrawing of effort and commitment from a goal	Be present, but no longer participate in the activities	.70	Going on vacation is too strenuous, efforts outweigh the pleasure	1.00	Consider moving to an apartment	.76
Re-engagement of goals	Identification, committing to and starting to pursue new goal(s)	Start taking pictures instead	.79	Make day trips	1.00	Eventually let the garden run wild and make a photo diary of it until I die	1.00

^a^Cohen’s kappa

## Results

### Part 1: results of vignette development

#### Content of the vignettes

The 11 vignettes all have a main character that is diagnosed with RA. In each situation, the impact of the disease on daily life is described as the main character always encounters a limitation or difficulty. The stories are set in three different domains: the social domain, the leisure activities domain, and the independent functioning domain (Table 3). Topics of the vignettes in the social domain are activities with partner, children, family, and friends. In the leisure activities domain, the topics are sports, holidays, hobby, and volunteering. In the independent functioning domain, the topics are gardening, household tasks, and running errands. Seven vignettes are formulated in the same way for men and women, except the name of the main character is entered to match the gender of the respondent. Four vignettes contain various activities focused on more typical female or male activities.

**Table 3:** Overview vignettes

Domain	Vignette	Short description	Limitation because of RA
Social	Partner	Day walking with partner	Fatigue
	Children (men)	Mountain biking every sunday morning with sons	Physical pain
	Children (woman)	Day of shopping with daughters	Fatigue and problems with fine motor skills
	Family Day^a^	Family Day with games and sports	Physical pain
	Friends	Weekend away with friends. Cycling 1 day during the weekend	Fatigue
Leisure activities	Sports	Twice weekly tennis.	Physical pain
	Caravan holiday^a^	2 weeks a year on vacation with a caravan	Unavailability of adjustments
	Hobby (men)	Model trains	Problems with fine motor skills
	Hobby (woman)	Create your own gift cards	Problems with fine motor skills
	Volunteering	Assist two mornings in a nursing home	Physical pain, fatigue
Independent functioning	Gardening^a^	Working in the garden. Always do everything yourself	Physical pain
	Household tasks	Major activities in the household, such as window cleaning	Physical pain, heavy work
	Running errands	Twice weekly errands	Physical pain, fatigue

^a^Vignette used in Part 2

#### Face validity

Seven questions were used in order to assess whether people understood the vignettes and whether they were face-valid (Table 4). The median scores were four or five, which means that on average the participants *agreed* or *totally agreed* with the statements. All participants understood the stories and 97 % agreed or totally agreed with the statement that the stories were easy to understand. Another 83 % could empathize with the main character, while 13 % responded neutrally to that question. Over 90 % agreed or totally agreed with the statement, “*I found the stories realistic/recognizable.*” The impact of RA was recognizable to 94 % of the participants, and another 87 % found the impact of RA realistic.

**Table 4:** Vignette face validity and comprehensibility: median, SD, and frequencies

	Median	1	2	3	4	5
I have understood the stories	5				5	26
The stories were easy to understand	5	1			6	24
I was able to empathize with Pieter/Karin	4		1	4	13	13
I found the stories realistic	4			3	16	12
I found the stories recognizable	5			2	13	16
I found the impact of RA recognizable	4		1	1	14	15
I found the impact of RA realistic	4			4	17	10

1 = totally disagree, 2 = disagree, 3 = neutral, 4 = agree, 5 = totally agree

The spontaneous reactions to the vignettes supported the general picture that respondents found the vignettes clear and recognizable. Some stories were more in line with the patient’s own life than others. Few participants (*n* = 5) disliked the stories because the main topic was about the disease. For example, spontaneous reactions of participants were: “*No, not fun to read if you empathize with the main character, as she experiences increasing limitations due to RA. It is recognizable though”* and *“I think it’s never fun to read because it is about a disease. I’d rather not read it.”* Some participants (*n* = 7) did not reflect on their own situation as they read the stories, for example: “*No, I am too down to earth for that”* and *“No, as my own situation is already adjusted.”* In general, respondents liked reading the stories.

### Part 2: goal management strategies in response to arthritis-specific vignettes

#### Solutions given in response to the problems described in the vignettes

A total of 262 respondents completed the questionnaire, of which 194 provided one or more solutions to the problems described in the vignettes (74 %). In total 1221 responses were given to the three vignettes (Table 5). One-third of the solutions submitted in response to the vignettes could be coded as the strategy maintenance of goals (30 %), closely followed by the strategy adjustment of goals (29 %). Another 21 % of the solutions were coded as disengagement of goals, where only 10 % involved the strategy re-engagement of goals. Another 10 % of the answers were unclassifiable, mostly ranging from comments on the applicability of the vignette (for example, “*I am still able to do this,*” and “*I have no garden*”) to answers showing that the instructions were not well understood (for example: “*I would maybe try this,*” and “*Yes, a lot of pain*”). In a minority of the unclassifiable responses, two themes were recognizable, i.e., stigma and positive recommendations, that did not relate to threatened goals, though they are related to arthritis.

**Table 5:** Frequencies, preferences, and distribution of the responses to the vignettes

Goal management strategy	Vignette 1 Family Day				Vignette 2 caravan holiday				Vignette 3 gardening				Total		
	Number of responses (%)	Median^a^	IQ range^c^	25–75 percentile	Number of responses (%)	Median^a^	IQ range	25–75 percentile	Number of responses (%)	Median^a^	IQ range	25–75 percentile	Number of responses (%)	Median^a^	IQ range
Maintenance	90 (19)	1	1.25	1–2.25	148 (36)	1	1	1–2	180 (38)	1	1	1–2	418 (31)	1	1
Adjustment	130 (28)	1	1	1–2	147 (35)	2	1	1–2	113 (24)	2	1	1–2	390 (29)	1	1
Disengagement	93 (20)	3	4	1–5	59 (14)	4	4	1–5	127 (27)	2	3	1–4	279 (21)	3	3
Re-engagement	122 (26)	1	1	1–2	11 (3)	2	3	1–4	1 (0)	2	0	2–2	134 (10)	1	1
Unclassifiable	33 (7)				51 (12)				47 (10)				131 (10)		
Total number of responses per vignette^b^	435				365				421				1221		

^a^1 = I would *absolutely* try this, 2 = I would *probably* try this, 3 = I would *maybe* try this, 4 = I would *probably not* try this, 5 = I would *never* try this

^b^Respondents could give a maximum of six responses

^c^IQ range, interquartile range

#### Preference of the goal management strategies

In general, participants would *absolutely* or *probably* try the solutions that they named. Only solutions that involved the disengagement of goals were less preferred, and on average, participants indicated that they would only maybe execute such disengagement solutions.

#### Strategies per domain

In the social domain (vignette 1), almost one-third of solutions suggested adjustment of the goal by participating less fanatically in the games and activities during Family Day (Table 5). Solutions coded as re-engagement were mentioned in 26 % of the answers; most people thought of joining the Family Day organization or becoming game judges. Maintenance of goals could be recognized in one-fifth of the answers, for example, when people suggested devices and tools that would facilitate participation in the games or that they would participate despite problems or pain later. Solutions coded as disengagement of goals contained, for example, skipping the day activities and only going for dinner and being there all day, but not taking part in the games. Solutions that involved the adjustment of goals, the re-engagement of goals, and the maintenance of goals were highly preferred. Solutions that entailed the disengagement of goals were less preferred.

In the leisure activities domain (vignette 2), solutions coded as maintenance of goals were mentioned most frequently. For example, most people mentioned the use of assistive devices or other adaptations to the environment to facilitate their stay in the caravan. Maintenance of goals was closely followed by the adjustment of goals, where people suggested arranging their holiday in a different way, for example, by staying in a holiday house or hotel instead of a caravan. Examples of solutions involving the disengagement of goals were: staying at home and selling the caravan. A small portion of the solutions involved the re-engagement of goals. For example, one solution was to take day trips instead of going on a two-week holiday. In this leisure activity domain, the solutions that involved the maintenance of goals were the highest preferred, followed by adjustment of goals and then re-engagement of goals. Solutions coded as disengagement once again had the lowest preference score.

In the domain-independent functioning (vignette 3), most solutions that people provided were coded as maintenance of goals. Solutions were, for example, to use assistive devices or to spread the gardening work over several days. The disengagement of goals was reflected in almost one-third of the solutions, for example, when respondents suggested having the garden completely maintained by a gardener or moving to an apartment. In one-fourth of the solutions, adjustment of goals was recognized, for example, when respondents suggested hiring a gardener or asking for help from family members for larger gardening tasks. Only one solution could be coded as containing re-engagement of goals, namely to “*eventually let the garden run wild and make a photo diary of it until I die.*” The solutions coded as maintenance of goals were the highest preferred, followed by adjustment of goals and disengagement of goals.

#### Themes and domains mentioned in the open vignette

A number of themes could be identified in the open vignette. In the vast majority of the answers, people reported about their *own limitation*s (e.g., pain, fatigue, functional limitations, or activities that they are no longer able to perform), *personal goals* that are threatened (e.g., an abandoned or threatened hobby or the personal solution for a threatened hobby), and their *course of disease* (adjustments already performed, thoughts about the future, precise disease course). In addition, two minor themes were recognized that not directly related to threatened goals. Firstly people described the *stigma* they experienced (specific experiences or, in general, a lack of understanding from others). Secondly respondents described *positive recommendations* (e.g., ways to stay positive, advice for functioning or how to stay independent). In a none-of-the-above category, descriptions of problems that were not directly related to arthritis or answers that expressed no problems with arthritis were grouped together.

## Discussion and conclusion

Our overall objective was to study domain-specific goal management in arthritis patients. In the first part of the study, 11 vignettes—situation-specific hypothetical stories in which the main character encounters a problem with a valued goal due to arthritis—were developed. The vignettes were found to be face-valid, that is, respondents found the situations and the impact of arthritis described in the vignettes understandable, realistic, and recognizable.

The second part of the study focused on the solutions given by patients with polyarthritis to resolve situations described in a subset of the vignettes. The goal management strategies, including goal maintenance, goal adjustment, goal disengagement, and goal re-engagement, were recognized in a large majority of the solutions. Only 10 % of the solutions could not be coded as one of the four pre-defined strategies. No new or other goal management strategy could be recognized in these unclassifiable answers, however, two types of responses clearly emerged. The first type consisted of comments on the applicability of the vignettes, and the second type was composed of comments showing that respondents did not understand the instructions. From these results, it can be concluded that the four strategies are exhaustive in response to the vignettes. This outcome supports the use of the integrated model of goal management in examining goal management in arthritis patients.

Overall, the strategies of goal maintenance, goal adjustment, and goal disengagement were frequently mentioned in all three domains. However, some differences in mentioned and preferred goal management strategies could be identified between the domains. While goal re-engagement was mentioned as a solution in a quarter of the responses to the social vignette, this strategy was rarely mentioned in response to the other two vignettes. The most popular strategies in the social domain were goal adjustment, i.e., still participating but less fanatically, and re-engagement, i.e., assuming another role in the event, for example by joining the organizing committee. On the other hand, maintenance of goals was less often mentioned in the social vignette in comparison with the other two vignettes, perhaps because adjusting goals and re-engaging in new goals were seen as acceptable alternatives in this particular vignette. Limitations in the social domain can provoke an increase in depressive symptoms [41] which may explain why people devise many different ways in order to remain involved in a social activity like a Family Day, either by scaling down or by searching for alternative social goals. In contrast, both in the leisure domain and the independent functioning domain, maintaining goals by customizing the environment and using assistive devices was most popular. Goal disengagement was mentioned in all three vignettes, but overall less preferred than the other strategies. One possible explanation for the unpopularity of disengagement is that the striving for personal goals is important for well-being and identity [39, 40]. It seems that people would rather try to adapt their personal goals than disengage from them despite serious limitations or problems that they might face when attempting to achieve the goal.

Earlier research revealed positive relations of adjusting threatened goals with the well-being of patients with arthritis [4]. Also for maintaining goals and re-engagement in goals, clear positive relations to successful adaptation were found [4, see also 49]. The main conclusion of the study of Arends et al. [4] was the importance of flexibility in the management of goals. The present study showed that people could come up with various strategies in their solutions. Future studies should reveal how people who experience threatened goals due to arthritis select and apply goal management strategies and how effective those strategies are for them.

An additional open vignette was used to study in an explorative way the themes and domains people might mention. An open vignette can also be seen as a way to receive feedback on the completeness of the domains in the set of vignettes developed in Part 1 of this study. From the analysis of the topics mentioned in the open vignettes, it appeared that people did not find any specific domain lacking from the developed vignettes. In fact, the functional limitations and domains mentioned by the participants corresponded to the content of the complete set of vignettes developed in the first part of this study. Therefore, we concluded that our set of vignettes is exhaustive. Two minor themes that were mentioned were similar to themes found in other studies, that is, firstly some respondents described experienced stigma by others [2, 51, 52], and secondly, respondents mentioned keeping positive as a recommendation to other patients [30, 53]. Those two themes also appeared in the unclassifiable answers to the first three vignettes. Obviously, these themes are important for a number of respondents.

Some critical comments can be given on the study. First is the absence of the work domain in the present set of vignettes. Clearly the (in)ability to work can be an important factor for arthritis patients, as problems with work due to arthritis can negatively influence quality of life [54], family income, status, and the availability of social support [28]. However, since employment status among polyarthritis patients greatly differs, it was difficult to develop a work-related vignette that would be recognizable to the majority of intended respondents. It would be worthwhile in future research to develop a vignette on full-time work for the subgroup of respondents that are working full time.

During the development of the codebook, it became clear that precision of recognition of goal management strategies was closely related to a clearly defined goal. For example, in the Family Day vignette, the threatened goal was ambiguous, and therefore, some answers were difficult to interpret and code. This shows that despite the use of vignettes, some lack of clarity unfortunately still exists with regard to the goals people had in mind when answering. Consequently, future studies should clearly define the threatened goal in the vignette and ask respondents already in the development process—for example, via cognitive interviewing techniques—for their interpretation of the threatened goal in the story.

In addition, the content of the Family Day vignette may not be representative of all social situations. The presented threatened goal in this vignette was not the quality of social relations, but rather the participation in a social activity. This should be kept in mind when interpreting the results of this study. Also, a selection of three vignettes was used to study their applicability with a large sample of patients. It is possible, therefore, that some respondents could not identify with the chosen selection. Future studies could use all the vignettes, in order to study more domain-specific goal management in patient populations. We chose not to analyze the given solutions per person, but to study the general patterns of strategies named by all the respondents. The responses of people who provided the maximum of six solutions thus counted more heavily than those who reported a smaller set. However, we were interested in general patterns and not in preferences for goal management strategies per individual.

Further research could offer more insight into the roles that both personal traits and characteristics of the situation play in the deployment of goal management strategies. Also, one can imagine that people in one life stage are rather more inclined to release goals in certain domains than people in other life stages. Similarly, people with severe functional limitations possibly make different choices than people who experience less limitations or disease severity. The vignettes can be a useful method for future research into differences in domain-specific goal management between groups of respondents. Further studies should focus on the predictive value of the vignettes for successful adaptation. Likewise, a comparison between dispositional questionnaires and domain-specific vignettes will give insight into the construct validity.

The developed vignettes can be used to study how arthritis patients cope with threatened goals in specific domains from a patient’s perspective. The vignettes were found to be face-valid, and the replies to the vignettes could be coded using a codebook. The use of a detailed codebook made it possible to apply the vignettes to a large sample of respondents. Responses to the developed vignettes provided valuable information about domain-specific goal management. Results showed that the preferences for goal management strategies differ per domain, emphasizing the importance of the addition of a situation-specific instrument. Finally, this study showed that using vignettes for measuring domain-specific goal management is a valuable addition to the existing questionnaires that measure dispositional goal management.
